# Creating a Healthy Life for the Elderly through Participation in Self-Media: A Study on the Demands of the Elderly in Self-Media

**DOI:** 10.3390/ijerph191912774

**Published:** 2022-10-06

**Authors:** Yo-Wen Liang, Jeng Wang, Shu-Ping Yu, Jin-Kwan Lin, Allan Chung

**Affiliations:** 1 Department of Industrial Design, National Taipei University of Technology, Taipei 10608, Taiwan; 2 Department of Nursing, Chang Gung University of Science and Technology, Taoyuan 33303, Taiwan; 3 Department of Industrial Engineering and Management, Ming Chi University of Technology, New Taipei 24301, Taiwan; 4 Department of Business and Management, Ming Chi University of Technology, New Taipei 24301, Taiwan

**Keywords:** the elderly, health care, self-media, Kano model, social media

## Abstract

In recent years, with the trends in digital media and a shift in the sources of information, self-media has gradually become a unique new type of media with considerable potential. Numerous related studies have also indicated that participating in self-media positively impacts the elderly, especially in self-media regarding healthcare and welfare. However, research has seldom explored the demands and services for elderly participation in self-media. In this study, the research targets were 55–75 years of age, in good health, with a monthly disposable income of more than TWD 30,000 (*N* = 180). The research methods had two aims: (1) to analyze the current well-known self-media and websites related to healthcare; and (2) via the Kano Model questionnaire, to survey and explore the demand for self-media among the elderly. The results summarize and describe the preferred layout, content items, interaction methods, and information display of self-media content for the elderly. We anticipate designing a self-media website platform that meets the demands of the elderly and that continues to develop into social media platforms and audio-visual content in the future.

## 1. Introduction

With the rapid development of the Internet and the continuous evolution of multimedia forms, the sources of information received and obtained by human beings in terms of food, clothing, housing, transportation, education, and entertainment have gradually shifted from television and radio to self-media and Livestream, and to various forms of online media [1]. These online media are diverse and can mainly be divided into websites, video platforms, social media, and other categories [2].

In terms of website media, famous brands in Taiwan such as WordPress, Wix, and Weebly, which are based on their website media services, allow users to build their own website content and design layouts, and build personal brand websites with a high degree of freedom. If users do not have professional knowledge of programming and design aesthetics, they can choose BSP-oriented (Blog Service Provider) blogging services, such as Google’s Blogger and Pixnet (Taiwan’s largest blog platform), as well as services such as ZiMedia funded by Hon Hai Group Fuying Data [3]. In terms of video media, YouTube, which Google bought in 2006, is still the most used video and audio platform in the global market, including Taiwan. Free and professional live broadcast software, such as Open Broadcaster Software (OBS), which can directly convert computer images into live webcasts, is also active in the video media market [4]. Finally, there is social media, which is now a part of the modern lifestyle and easily accessible to the public. Social media generally occupies a significant amount of time in our lives and it has become an essential tool for interpersonal communication and connection, for example, Facebook, Line, Instagram, Twitter, and other brand services [5]. At present, through the construction and operation of self-media, a company’s brand image, business philosophy, product and service characteristics, and daily life can be expressed through website media with text and image expression capabilities and communicated dynamically through video media [6]. Additionally, a company can interconnect and communicate with all users’ social media to attract target groups and potential groups for marketing and communication purposes [7]. Finally, the whole process allows enterprises to increase opportunities to network with the public, increase interaction with netizens, and gain an in-depth understanding of the medium of demand.

However, owing to the burgeoning of Internet technology, digital brand services and advertising dissemination have gradually changed in the media sector [8,9]. Under this trend, after 2005, widespread construction and design of the content of websites, video platforms, and social media allowed the original online media to spread information to readers in one direction only. Following the web 1.0 era, everyone could create their own online media content and interact with the media created by others, officially entering the web 2.0 era with information interconnection and two-way communication [10]. Changes in the media toward action, real-time, diversification, and thinning have meant that the media plays a significant role in regard to lifelong learning [11]. According to a survey report by the National Development Council of Taiwan, 98.2% of people have used wireless or mobile Internet access, and 42.8% of people over 65 have Internet experience. The trend is to maintain growth, and there is also great potential for development [12]. In addition, using social media positively affects the elderly, allowing them to interact with old friends and new friends across the ages in real-time, to share their rich life stories, and find new activities and interests [13].

### 1.1. Definition of Self-Media

Self-media is a term derived from the notion of media independence and multi-point development driven by Internet technology. It is defined as an individual account operated through Internet technology and media, which publishes information and content to the world [14]. Through the management of self-media, a brand’s products or services are able to completely dominate the content information and media forms that it produces on the Internet, which is an essential task in modern Internet marketing [15]. Moreover, under the current prevalence of self-media, online marketing is considered almost equivalent to the business strategy of self-media. As outlined above, self-media can be used to communicate a company’s brand image, business philosophy, product and service characteristics, etc., through website media by using text, images and video media to create dynamic communication and interconnection [16]. All users’ self-media, and other methods, can be used to target potential groups for the purpose of marketing and communication. Self-media can also create opportunities for enterprises to increase their contact and interaction with the public and understand their needs. Below is the ranking of the top ten sites with the highest online media traffic in Taiwan by Alexa [17], as shown in Table 1.

### 1.2. Development of Self-Media

Whether it is a brand of physical products or virtual services, the first step in establishing self-media is to have a main website that can be used for information introduction, content publishing, and systematic page structure, that is, an official website [18]. The website is the only platform that can currently integrate all media forms in the self-media because of the HTML syntax structure of the website and the use of programming languages such as Cascading Style Sheets (CSS) and JavaScript [19]. Furthermore, the website can integrate text, pictures, videos, interactions, and even media content such as VR and AR displays, which are very suitable choices as the leading content platform for self-media [9]. In addition, the website is the only platform that can integrate various media services, such as embedding video content into the website (e.g., YouTube) through grammar and linking social media interactive messages back to the website display (e.g., Facebook).

Taiwan’s self-media platforms can be categorized as traditional websites or Blog Service Providers (BSP). Users can take advantage of their flexibility to build an independent and no advertisement website; however, this kind of website might need to pay for a website address, rent server space and successive traffic flow, and/or require knowledge such as HTML, CSS, and JavaScript [20,21], and the ability to write and edit programming syntax. The advantage of BSP is that it is easier to build content. Most servers have a complete article publishing system that provides free services [22]. Nevertheless, the disadvantage of BSP is that the website has insufficient layout and editing rights, which often appear on the layout. The service provider’s advertisements take up space, and when the site needs to move, it is not that easy to use; moreover, SEO is counted on the service provider’s domain, which does not proceed with the users.

### 1.3. Demand Research Methods for Self-Media

Once the platform, construction, and content of self-media are completed, most of them will conduct usability testing and evaluations to correct any self-media problems and maintain the conditions for easy use. Among them, the most widely used method for evaluating usability is usability engineering [23,24,25]. Nielsen (1993) believes that the most important indicators of usability are easy to use, efficient to use, easy to remember, subjectively pleasing, and few errors. When the indicators of usability engineering can be used to design the evaluation scale, experts and researchers in related design fields are invited to participate in the scale’s evaluation. Finally, the results are verified by statistical methods to assess whether the performance is significant [23,25]. The five essential indicators of usability engineering are shown in Table 2.

### 1.4. The Health Effects of Self-Media on the Elderly

Numerous studies have found that self-media positively affects the physical and psychological health of the elderly. A few years ago, Dogruel, Joeckel, and Bowman [26] expanded approaches to technology acceptance and use by developing a model to explain the entertainment-related benefits of new media technology for the elderly. Green, Tesler, and Sharon [27] noted that during the pandemic, self-media and social media played a vital role in providing online health information to the elderly. Specifically, reliable information encourages the elderly to access self-media and social media. Kim, Kang, and Park [28] proved in their study that therapeutic recreational media and programs for the elderly can be an effective way to introduce positive changes. Although many studies have pointed out that self-media and social media have positive effects and influences on multiple aspects related to the elderly, few studies have been devoted to researching user preferences and the needs of the elderly in regard to self-media.

Therefore, the authors of this study believe that based on the above research, exploring the needs of the elderly in regard to self-media will help to provide the elderly with a positive experience in using self-media, and at the same time, it can help them to achieve mental and physical health and happiness. On the other hand, regarding professional experience exchange and wisdom inheritance, the elderly and related service personnel can be trained to use self-media to share and discuss various healthcare experiences to promote the health and richness of the life of the elderly, and achieve the win-win goal of physical and mental balance.

## 2. Methods

### 2.1. Standards for Selection of Research Target

In this study, the research targets were 55–75 years of age, in good health, and with a monthly disposable income of more than TWD 30,000 (referring to the disposable amount after deducting daily expenses) or they owned immovable property. Participants were recruited via the snowball sampling method because of the convenience of sampling and to find suitable elderly participants (snowball sampling is a non-probability sampling technique). According to the IRB, we had to recruit participants who accept self-disclosure during the questionnaire survey. Therefore, the participants were asked to introduce other subjects to answer this questionnaire via snowballing, which improves self-disclosure and willingness to respond. The tasks were conducted by four trained interviewers between March and September 2020. All participants provided their contact information; thus, corrections could be made by phone or email and we could ensure no missing data at the end. The final recruited sample size was 192 for the questionnaire survey, and the number of valid questionnaires was 180. All the procedures in this study were performed following the guidelines of the 2013 World Medical Association Declaration of Helsinki. They were approved by the Research Ethics Committee of Chang Gung Medical Foundation Institutional Review Board in Taiwan (IRB number: 201811081999-03).

### 2.2. Research Procedure

#### 2.2.1. Analysis of Existing Well-Known Healthcare Self-Media

**Figure 1:**
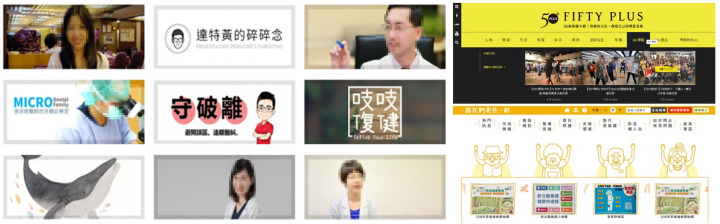
Analysis of existing well-known healthcare self-media.

By analyzing current, well-known and popular self-media websites related to healthcare on issues related to the elderly, this research parsed the content of self-media websites that were relevant to the needs of the elderly. This study analyzed the following well-known health--related self-media websites, including (1) Fang Miao’s essays; (2) Dart Huang’s thoughts; (3) Dermatologist, Dr. Lin Zhengxian; (4) Dr. Zhang Tianhao’s dentist office; (5) Da Ren Medical correction; (6) I am Shirley, an enthusiastic Psychologist Physician; and (7) Li Ai-lin TCM Physician et al. References to related websites for the elderly include 50plus—we are old together. The splash screen page is shown in Figure 1.

#### 2.2.2. Questionnaire Design to Study the Elderly’s Self-Media Demands

The questionnaire used in this research comprised two parts: the basic information about the respondents and the items regarding self-media demands. The basic characteristics of the respondents include gender, age, education, preference for self-media, the time of self-media use, the way they surfed self-media, the most concerning issues on self-media, and descriptive statistics about the users’ orientation and the characteristics of the participants. The items measuring self-media demands included the demands with regard to the construction of the self-media form, self-media content, self-media interactive mode, and self-media information display requirements.

The questionnaire items regarding self-media demands was designed based on the results of the analysis of existing well-known healthcare self-media (see Section 2.2 above). In addition, the design structure of this part of the questionnaire utilized the Kano model as the method. The Kano model was proposed by Dr. Noriaki Kano, a professor at the Tokyo Institute of Technology, Japan [29]. Applying the Kano Model in the questionnaire allows participants to analyze the questions via a two-dimensional forward and reverse thinking modem and six different classification results for demand items or satisfaction items [30].

**Figure 2:**
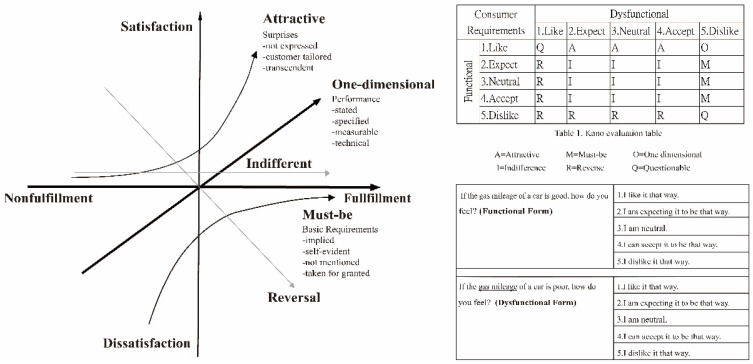
The Kano Model’s theoretical framework and an example of the questionnaire design method.

Three levels of demand quality can be obtained from the Kano Model questionnaire: the first stage provides the most basic requirements acceptable to customers (quality control), and the appropriate demand level is the must-be quality element (M); the second stage is to meet the needs of customers (quality management), and the level of related demand is the one-dimensional attribute (O); the third stage is to meet the potential needs of customers to achieve customer satisfaction, which is called attractive quality creation, and the level of related demand is the attractive attribute (A). The theoretical framework of the Kano model and an example of the questionnaire design are shown in Figure 2.

Therefore, the Kano model can calculate and evaluate the level of the participants’ design requirements or explore potential needs. Because the elderly may be reserved or cautious, simple research questionnaires may not be able to extract real answers and their thoughts. However, the Kano model questionnaire is very suitable for exploring the needs of this group of people.

#### 2.2.3. Kano Questionnaire Survey to Explore the Elderly’s Self-Media Demands

The researchers visited Taiwan’s Taoyuan Chang Gung Health Village, Yunlin Mai-Liao Six Industrial Zone, Kaohsiung Formosa Heavy Industry Ren-Wu Factory, and other institutions to find suitable participants. Hence, this research used data from these institutions; furthermore, the data could be used in the following stage.

## 3. Results

### 3.1. Results of Well-known Healthcare Self-Media Analysis

Based on the results of the well-known healthcare self-media analysis in Taiwan, this study determined the self-media items that were preferred by the elderly and related to the demands of the elderly as follows: daily life, issues, video sharing, finance, innovation and retirement, expert columns, about us, announcements, news, event calendars, overview, courses, health knowledge, picture carousel, volunteers’ party, sports’ party, Q&A, subsidy information, friendly space, hospital information, 50+ colleges, latest articles, Facebook, YouTube, Line@, Instagram, popular articles, popularity, number of visitors, etc.

Further in-depth analysis of the details highlighted the construction of self-media related to medical care. Different design elements and display methods can be further considered in terms of form, content, interaction method, information display, etc. The questionnaire content can be based on a summary of the design and creation.

#### 3.1.1. Self-Media Construction Form Demands

**Figure 3:**
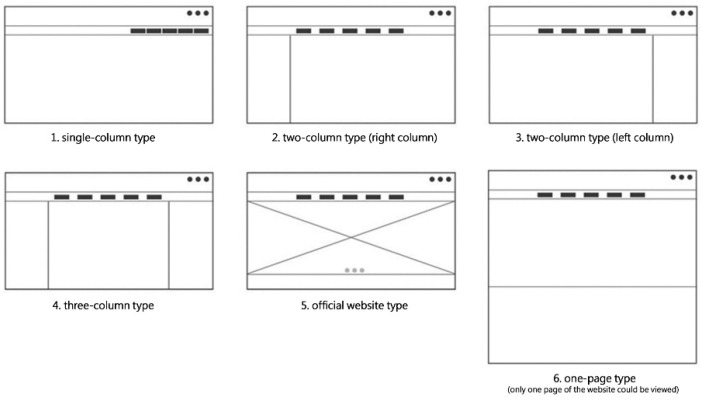
The self-media construction forms in the Kano questionnaire.

According to the analysis of several Taiwanese healthcare-related self-media concerns of the elderly, this study found that the common construction forms were: (1) single-column type; (2) two-column type (right column); (3) two-column type (left column); (4) three-column type; (5) official website type; (6) one-page type (only one page of the website could be viewed). The six main types of website media are shown in Figure 3 below.

#### 3.1.2. Self-Media Construction Content Demands

According to the analysis of several Taiwanese healthcare-related self-media concerns of the elderly, this study found that the typical content comprised: about us and our story, news and live current affairs, senior life experience, sharing of great people, party or fun event calendar, innovative elderly care issues, latest elderly care case studies, aged living aesthetics, volunteer work activities, sports activities, course overview, healthcare knowledge content (health preservation, Taijiquan, health and medicinal knowledge, etc.), senior life financial management, life-related (knowledge, things suitable for the elderly), elderly-friendly spaces (a place match for the elderly), video and audio (healthcare, play, elderly issues), hospital-related information (the latest hospital information, information from partner hospitals), font size and small size Toggles, news and life current affairs, links to interviews with audio-visual characters, stories about us, column content (related associations and people), etc.

#### 3.1.3. Self-Media Construction Interaction Method Demands

According to the analysis of several Taiwanese healthcare-related self-media concerns of the elderly, this study found that the standard interaction methods of concern were: leave a message, reply to articles, send messages to someone, chat with others online, search for articles and information, collect articles, bookmark favorite articles or other items, etc.

#### 3.1.4. Self-Media Construction Information Display Demands

According to the analysis of several Taiwanese healthcare-related self-media concerns of the elderly, this study found that the common interaction methods were: time display, visitor number statistics, online population number display, popularity display of the day, latest article display, links to other media (ex. Facebook/YouTube/Line), popular articles display, links to other related information outside the site, website- related frequently asked questions and other items, etc.

### 3.2. Results of the Descriptive Statistics for Participants (Elderly)

**Figure 4:**
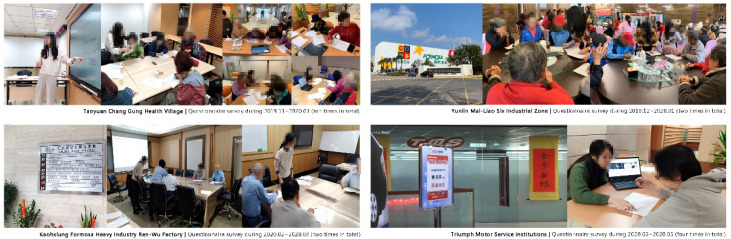
The implementation process of the questionnaire survey.

In this study, a total of 198 participants were surveyed through the actual distribution of paper questionnaires (the age group is between 55 and 75 years of age and the monthly disposable income exceeds TWD 30,000). The number of valid questionnaires returned was 180. There were 18 invalid questionnaires (the Kano model could determine the validity of the questionnaire and invalid questionnaires with contradictory results were excluded); thus, the actual valid questionnaires totaled 180. The researchers in this study visited Taiwan’s Taoyuan Chang Gung Health Village, Yunlin Mai-Liao Six Industrial Zone, Kaohsiung Formosa Heavy Industry Ren-Wu Factory, and Triumph Motor Service institutions to conduct the survey over a total of six months, as shown in Figure 4 below.

**Figure 5:**
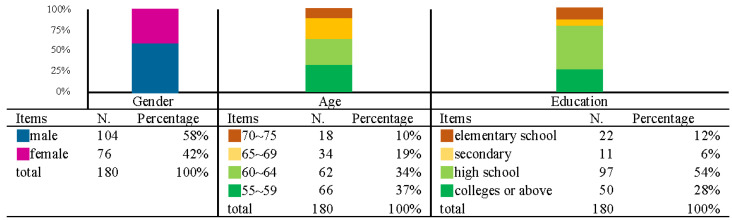
Descriptive statistical results of participants’ gender, age, and education.

According to the descriptive statistical results of the primary data of the participants, the first part was the analysis of gender, age, and education. Among the participants surveyed, 58% were male and 42% female who were mainly aged between 55–59 years (37%) and 60–64 years (34%), and the education level included 54% high school vocational students with 28% college and university students as the majority. The descriptive statistical results of participants’ gender, age, and education are shown in Figure 5.

**Figure 6:**
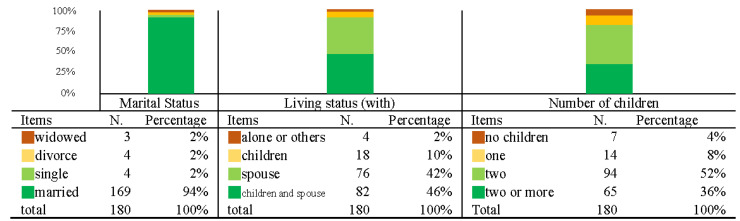
Descriptive statistical results of participants’ marital status, living status, and the number of children.

The second part analyzed marital status, living status, and the number of children. Among the participants surveyed, the proportion of the elderly who were married accounted for 94% and the proportion of the elderly living with children and a spouse and with a spouse accounted for 46% and 42%, respectively. The number of elderly living with two children showed the highest percentage (52%) and second was the proportion with more than two children (36%). The descriptive statistical results for participants’ marital status, living status, and the number of children are shown in Figure 6.

**Figure 7:**
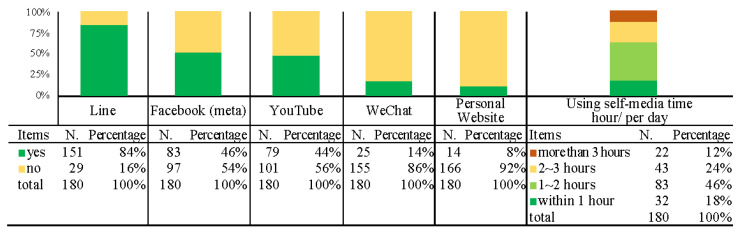
Descriptive statistical results for participants’ self-media use and frequency.

The third part is the descriptive statistical results of the participants’ experience using self-media, including LINE, Facebook (FB), WeChat, YouTube, personal website, and time spent on self-media. Among the participants surveyed, the proportion using LINE was the highest, as high as 84%, followed by 46% using Facebook and 44% using YouTube. Regarding the amount of time spent on self-media, the highest percentage (46%) spent 1–2 h per day. The descriptive statistical results for the self-media type used by participants and the frequency of use are shown in Figure 7.

**Figure 8:**
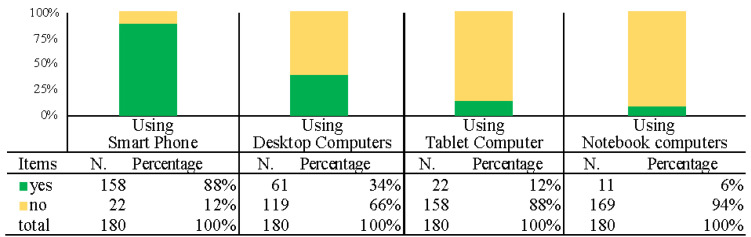
Descriptive statistical results for participants’ device selection for self-media surfing.

The fourth part includes the descriptive statistical results of the participants’ self-media surfing device selection, which analyzed the choice of self-media viewing device such as smartphones, desktop computers, tablet computers, and notebook computers. The results showed that 88% of the respondents use smartphones, the highest, followed by desktop computers at 34%, and all others were below 20%. The descriptive statistical results of participants’ device selection for self-media surfing are shown in Figure 8.

**Figure 9:**
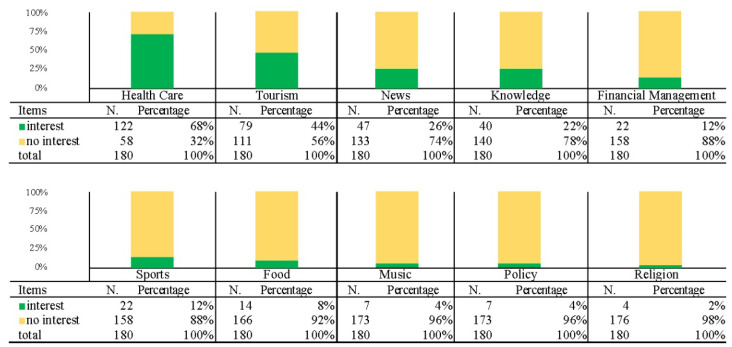
Descriptive statistical results of participants’ preferred self-media subjects.

The fifth part is the descriptive statistical results of the participants’ preferred self-media subjects. This part allows the participants to fill in the questionnaire in an open-ended manner. The results showed 10 subjects that the participants mentioned at least twice. Among the subjects, medical care accounted for the highest proportion with 68%, followed by tourism with 44%, news with 26%, knowledge with 22%, and sports and financial management with 12%. The descriptive statistical results of participants’ self-media preferred subjects are shown in Figure 9.

### 3.3. Results of Self-Media Demands via Kano Model Questionnaire for Participants (Elderly)

**Figure 10:**
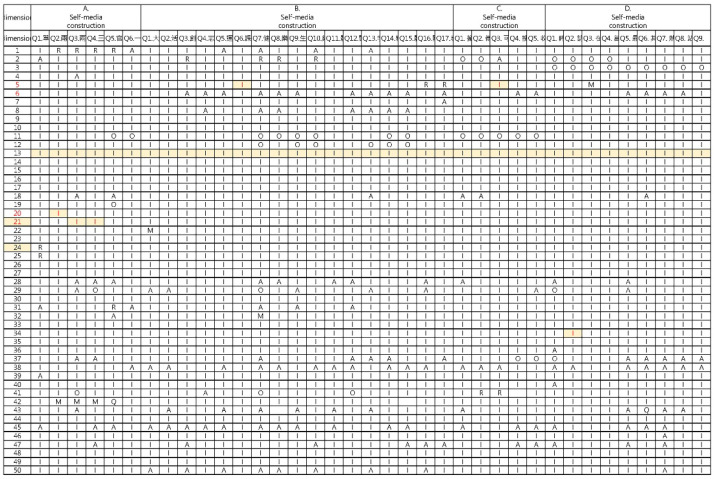
Kano model statistical process for self-media demand research.

This study utilized the Kano questionnaire to analyze the demand for 37 functional items (from the results of the analysis in Section 3.1) in four categories of demand. The four categories of requests enabled us to further explore whether the elderly’s demands for self-media belong to the attractive attribute (A), the one-dimensional need (O), the must-be quality element (M), the indifferent quality element (I), or the reverse quality element (R). The processing of the whole dataset by Excel calculation is shown in Figure 10.

The questionnaire items was converted into a frequency distribution table of Kano quality (A, O, M, I, R, Q) after all 180 valid questionnaires were evaluated and analyzed by the Kano model. Based on the analysis results, we can better understand the functional requirements of each aspect of feedback from the participants. According to the analysis of the research data, the four items of the questionnaire: the demands regarding the form of self-media construction, the demands regarding the content of self-media construction, the demands regarding the interaction methods of self-media construction, and the demands regarding the information display of self-media construction, and a total of 37 demand items classified by other statistical analysis was performed, and the results are shown in Table 3.

In the next stage, we further calculated the needs evaluation (A, O, M, I, R, Q), Satisfied Impact (SI), and Dissatisfied Impact (DSI) after converting the frequency distribution table into percentages. According to the results of the statistical analysis, the must-have quality elements (M) are A-Q5, official website type; A-Q6, one-page type; BQ2, party or fun event calendar; B-Q6, lifelong learning courses and information; B-Q8, article related to senior living; C-Q1, leaving a message and replying to articles; C-Q3, chat with others online; D-Q1, time display; D-Q2, visitors’ statistics; D-Q5, the latest article; etc. When developing self-media dedicated to the elderly, these functional requirements must be listed in the design list. In addition, the attractive attribute quality elements (A) are A-Q1, single-column type; B-Q11, elderly-friendly video; B-Q14, keynote speech video; C-Q5, collecting favorite articles; D-Q7, popular articles; etc. The attractive attribute quality functions do not trigger negative impacts but improve users’ desires for the product. Therefore, they can be used as unique features and main functions during self-media development.

However, it should be noted that the reverse quality elements (R) were A-Q4, three-column type; B-Q1, great people sharing experience; B-Q15, keynote speech video; D-Q9, Q&A; etc. Self-media satisfaction will decrease significantly if these reverse requirements are developed and designed. Other elements belonging to the indifferent quality category (I) have no positive or negative impact on requirements and satisfaction after development and design. The evaluation results of the Kano model questionnaire for functional items in this study are shown in Table 4.

In the last stage, this study imported the Satisfied Impact (SI) and Dissatisfied Impact (DSI) for each functional requirement item into the sensitivity matrix of the Kano model. Sireli, Kauffmann, and Ozan [31] demonstrated that the Kano weight calculation method integrates SI and DSI. By comparing the satisfaction index (SI) and the dissatisfaction index (DSI), researchers assume that the satisfaction and the dissatisfaction has the same weight on the target group. That is, within the standard radius range of the sensitivity matrix, the sensitivity of quality characteristics is not enormous. The importance of the sample depends on the origin and its relative position in the coordinate: the closer, the ignored and the farther, the more prioritized.

**Figure 11:**
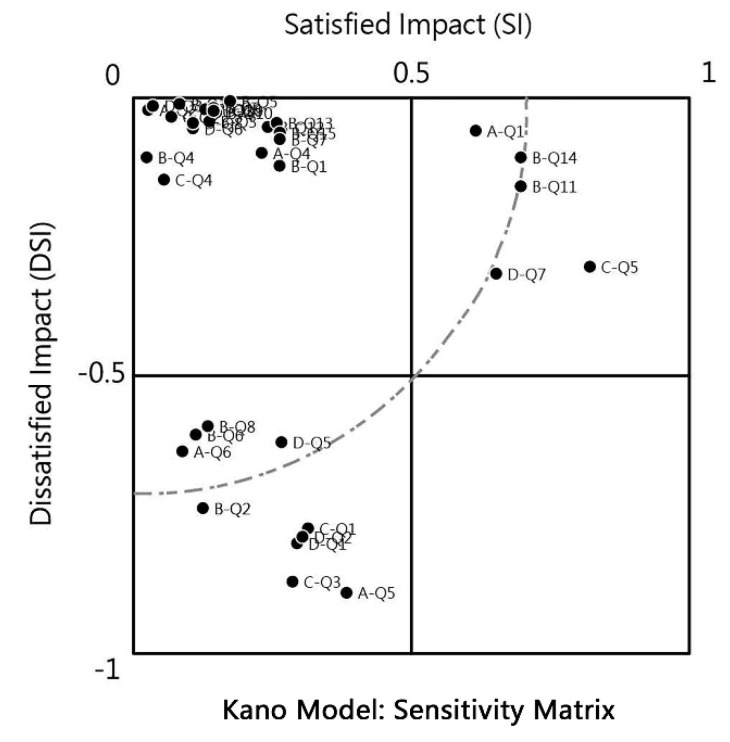
Sensitivity matrix of Kano model questionnaire for functional items.

Based on the results of the sensitivity matrix, the items of A-Q5, official website type; B-Q2, two-column type (right column); B-Q11, elderly-friendly video; B-Q14, news and life current affairs; C-Q1, leaving a message and replying to articles; C-Q3, chat with others online; C-Q5, collecting favorite articles; D-Q1, time display; D-Q2, visitors’ statistics; D-Q7, popular articles; etc., were the main demand items that are required to be prioritized in the development and design of self-media (distributed on the periphery of the sensitivity matrix). Moreover, several others could be considered as development and design items (distributed around the outer boundary of the sensitivity matrix), such as A-Q1, single-column type; A-Q6, one-page type; B-Q6, lifelong learning courses and information; BQ8, senior life financial management; D-Q5, the latest article. The sensitivity matrix for the Kano model questionnaire for functional items is shown in Figure 11.

## 4. Discussion

### 4.1. Discussion of Descriptive Statistics Results for Participants (Elderly)

Based on the descriptive statistics for gender and educational attainment (Figure 5), the study population (55–75 years of age and with a monthly disposable income of more than TWD 30,000) comprised slightly more males than females, but there was not a big difference. Regarding education level, the combined percentage of 54% for high school vocational colleges and 28% for tertiary institutions exceeded 80%, indicating that education will positively impact.

The second part analyzed the marital status, living status, and number of children (Figure 6). The proportion of participants who were married was as high as 94%, which means that almost all of the target group are married and remain married. Among them, 42% live with their partners, and 46% live with their children and partners, and the total exceeds 90%, indicating that these are the main living situations for most of the target group. The environment is an essential reference. The last item is the number of children, 52% have two children and 36% have more than two children; the total is more than 80%, indicating that most of the target group have children and more than one child. This phenomenon is very different from the current situation.

The descriptive statistical results regarding self-media use (Figure 7) show that the current target group uses LINE (as high as 84%), while Facebook (46%), and YouTube (44%) are used by less than 50%, while the use of other self-media is limited. These statistical results demonstrate that there is still much space for the improvement and development of self-media in expanding the target group.

The fourth part was the descriptive statistical results regarding self-media viewing methods (Figure 8). According to the data, the target group mainly use smartphones (88%) as the main viewing method, followed by only 34% who use desktop computers. When developing self-media for use by a target group, the primary consideration is the UI environment with the mobile phone interface, supplemented by computer web pages. The results were very different from several previous studies that point out that the elderly still use computers as the main viewing method when using media products. In addition, the situation could be further interpreted as indicating that the elderly are open to using the latest technology products, and are especially good at using mobile phones to participate in various self-media activities.

The last item of analysis was the target group’s subject preference and discussion on self-media (Figure 9). The only topic with more than a 50% preference was healthcare (68%), followed by travel (44%), with less than 30% for the others, indicating that the members of the target group still care about themselves. Health attracts the most significant concern and attention, followed by the desire for information on travel and learning about various places.

### 4.2. Discussion of Self-Media Demands via Results of the Kano Model Questionnaire for Participants (Elderly)

According to the evaluation results of the Kano Model questionnaire shown in Table 4 for the functional items, the items that meet the must-have quality demands (M) were AQ5, official website type; A-Q6, one-page type; B-Q2, party or fun event calendar; BQ6, lifelong learning courses and information; B-Q8, senior life financial management; C-Q1, leaving a message and replying to articles; C-Q3, chat with others online; D-Q1, time display; D-Q2, visitors’ statistics; and D-Q5, latest article. The demand items could be essential elements that must be incorporated when developing and designing functions. Moreover, using the items above combined with the sensitivity analysis matrix (Figure 11), we can further determine that the following items are important: A-Q5, official website reading media; B-Q2, event calendar content (party or fun); C-Q1, message and reply to articles; C-Q3, online chat and dialogue with other netizens; D-Q1, time display; DQ2, the total number of visitors, and other items. These also need to be regarded as essential items in the fundamental design and can be discussed in depth in future research. These programs should be designed to be more acceptable to the target group.

According to Table 4, the items that belong to the attractive attributes (A) are A-Q1, single-column type; B-Q11, the elderly-friendly video; B-Q14, news and live current affairs; C-Q5, collecting favorite articles; D-Q7, popular articles; etc. The abovementioned items also fall near the radius dotted line in the results of the sensitivity matrix in Figure 11. While these items may not be the most urgent demands, they could be introduced into the design of self-media, as this will significantly increase the target group’s satisfaction and interest in self-media.

## 5. Conclusions

With the gradual popularization of independent individual social media accounts via Internet technology and media, people can completely control the information and media forms.

Regarding self-media publications and creators, the elderly are very much in the minority, and thus there is considerable room for development. Many studies have also confirmed that self-media positively impacts and affects the elderly. In addition, some studies have shown that using media allows seniors to interact with old friends and new friends across the ages in real-time, share their life-long experiences, and relieve the monotony of daily life. The results of this research can be used as a follow-up to explore the needs and preferences of the elderly in terms of medical and healthcare, and then to design a self-media website that meets their demands and even continues to develop into social media and video platforms. We hope to fulfil the ultimate goal of the elderly participating in self-media with ease and enthusiasm.

## Figures and Tables

**Table 1:** The top ten sites with the highest media traffic in Taiwan.

Ranking	Site	Daily Time on Site (mm: ss)	Daily Page Views per Visitor	% of Traffic from Search	Total Sites Linking in
1	Google.com.tw	7:11	12.36	0.60%	14,214
2	Youtube.com	8:18	4.81	16.00%	3,099,355
3	Pixnet.net	3:15	2.38	67.90%	85,782
4	Facebook.com	10:11	4.08	8.70%	8,244,720
5	Google.com	7:50	8.91	4.80%	4,105,884
6	Yahoo.com	4:06	3.68	8.40%	775,444
7	Ettoday.net	3:59	2.50	22.00%	13,615
8	Ltn.com.tw	3:45	2.68	34.10%	12,506
9	Momoshop.com.tw	7:21	3.45	32.70%	9336
10	Setn.com	3:57	2.65	12.00%	3724

**Table 2:** Usability engineering index and explanation.

Usability Engineering Index	Indicator Description and Explanation
Easy to use	The platform is easy for users to learn and use. The self-media should be easy to understand and allow users to start working with the system or interface quickly.
Efficient to use	The platform should allow users to operate efficiently. The self-media should be accessible, and they can quickly get high performance.
Few errors	The platform should have low error rates for users. Additionally, the self-media should allow users to use it without making too many mistakes; even if they make mistakes, they can be quickly dealt with. Moreover, it must avoid fatal errors.
Easy to remember	The platform makes it easy for users to remember. The self-media or interface should be easy to remember for casual users who have not operated for some time and do not need to learn from scratch when they return to this system.
Subjectively pleasing	Users are satisfied with using the self-media. The self-media should be pleasant to use so that users are satisfied with the system.

**Table 3:** The frequency distribution table of the Kano Model questionnaire for functional items (*N* = 180).

Questionnaire Dimensions	Questionnaire Items	A (Attractive)	O (One-Dimensional)	M (Must-be Quality)	I (Indifferent Quality)	R (Reverse Quality)	Q (Questionable)	Total
A. Self-media construction form demands	Q1. single-column type	111	0	11	54	4	0	180
	Q2. two-column type (right)	3	0	2	172	3	0	180
	Q3. two-column type (left)	21	1	1	154	3	0	180
	Q4. three-column type	14	3	4	52	107	0	180
	Q5. official website type	22	51	102	2	3	0	180
	Q6. one-page type	8	6	105	59	2	0	180
B. Self-media construction content demands	Q1. sharing experience	15	2	4	48	111	0	180
	Q2. party calendar	17	2	125	36	0	0	180
	Q3. elderly care issues	27	1	2	148	2	0	180
	Q4. volunteer activities	17	0	2	161	0	0	180
	Q5. sports activities	24	2	2	151	1	0	180
	Q6. lifelong learning courses	15	3	104	58	0	0	180
	Q7. health care knowledge	40	9	3	127	1	0	180
	Q8. financial management	22	2	97	57	2	0	180
	Q9. article related to senior living	25	6	2	147	0	0	180
	Q10. the elderly-friendly space	26	3	3	146	2	0	180
	Q11. the elderly-friendly video	123	3	20	32	2	0	180
	Q12. hospital related information	33	6	3	137	1	0	180
	Q13. font size toggles	36	8	1	134	1	0	180
	Q14. news and life affairs	119	7	15	38	1	0	180
	Q15. keynote speech video	20	2	3	62	93	0	180
	Q16. stories about us	23	2	2	151	2	0	180
	Q17. Experts’ speech	13	1	2	162	2	0	180
C. Self-media construction interaction method demands	Q1. leaving a message and replying to articles	19	40	98	23	0	0	180
	Q2. messages sending	10	2	4	163	1	0	180
	Q3. chat with others online	11	52	107	8	2	0	180
	Q4. searching for articles	21	5	4	150	0	0	180
	Q5. favorite articles	121	27	30	2	0	0	180
D. Self-media construction information display demands	Q1. time display	24	36	108	12	0	0	180
	Q2. visitors’ statistics	34	35	101	3	7	0	180
	Q3. online population	4	3	3	170	0	0	180
	Q4. popularity display	6	3	2	169	0	0	180
	Q5. latest article	23	26	81	50	0	0	180
	Q6. links to other media	14	4	3	159	0	0	180
	Q7. popular articles	78	40	31	31	0	0	180
	Q8. related information	15	3	2	160	0	0	180
	Q9. Q & A	11	3	3	74	89	0	180

**Table 4:** The evaluation results of the Kano model questionnaire.

Questionnaire Dimensions	Questionnaire Items	A (%) (Attractive)	O (%) (One-Dimensional)	M (%) (Must-be Quality)	I (%) (Indifferent Quality)	R (%) (Reverse Quality)	Q (%) (Questionable)	Result of Evaluation	SI (Satisfied Impact)	DSI (Dissatisfied Impact)
A. Self-media construction form demands	Q1. single-column type	61.7%	0.0%	6.1%	30.0%	2.2%	0.0%	A	0.63	−0.06
	Q2. two-column type (right)	1.7%	0.0%	1.1%	95.6%	1.7%	0.0%	I	0.02	−0.01
	Q3. two-column type (left)	11.7%	0.6%	0.6%	85.6%	1.7%	0.0%	I	0.12	−0.01
	Q4. three-column type	7.8%	1.7%	2.2%	28.9%	59.4%	0.0%	R	0.23	−0.10
	Q5. official website type	12.2%	28.3%	56.7%	1.1%	1.7%	0.0%	M	0.41	−0.86
	Q6. one-page type	4.4%	3.3%	58.3%	32.8%	1.1%	0.0%	M	0.08	−0.62
B. Self-media construction content demands	Q1. sharing experience	8.3%	1.1%	2.2%	26.7%	61.7%	0.0%	R	0.25	−0.09
	Q2. party calendar	9.4%	1.1%	69.4%	20.0%	0.0%	0.0%	M	0.11	−0.71
	Q3. elderly care issues	15.0%	0.6%	1.1%	82.2%	1.1%	0.0%	I	0.16	−0.02
	Q4. volunteer activities	9.4%	0.0%	1.1%	89.4%	0.0%	0.0%	I	0.09	−0.01
	Q5. sports activities	13.3%	1.1%	1.1%	83.9%	0.6%	0.0%	I	0.15	−0.02
	Q6. lifelong learning courses	8.3%	1.7%	57.8%	32.2%	0.0%	0.0%	M	0.10	−0.59
	Q7. health care knowledge	22.2%	5.0%	1.7%	70.6%	0.6%	0.0%	I	0.27	−0.07
	Q8. financial management	12.2%	1.1%	53.9%	31.7%	1.1%	0.0%	M	0.13	−0.56
	Q9. article related to senior living	13.9%	3.3%	1.1%	81.7%	0.0%	0.0%	I	0.17	−0.04
	Q10. the elderly-friendly space	14.4%	1.7%	1.7%	81.1%	1.1%	0.0%	I	0.16	−0.03
	Q11. the elderly-friendly video	68.3%	1.7%	11.1%	17.8%	1.1%	0.0%	A	0.71	−0.13
	Q12. hospital related information	18.3%	3.3%	1.7%	76.1%	0.6%	0.0%	I	0.22	−0.05
	Q13. font size toggles	20.0%	4.4%	0.6%	74.4%	0.6%	0.0%	I	0.25	−0.05
	Q14. news and life affairs	66.1%	3.9%	8.3%	21.1%	0.6%	0.0%	A	0.70	−0.12
	Q15. keynote speech video	11.1%	1.1%	1.7%	34.4%	51.7%	0.0%	R	0.25	−0.06
	Q16. stories about us	12.8%	1.1%	1.1%	83.9%	1.1%	0.0%	I	0.14	−0.02
	Q17. Experts’ speech	7.2%	0.6%	1.1%	90.0%	1.1%	0.0%	I	0.08	−0.02
C. Self-media construction interaction method demands	Q1. leaving a message and replying to articles	10.6%	22.2%	54.4%	12.8%	0.0%	0.0%	M	0.33	−0.77
	Q2. messages sending	5.6%	1.1%	2.2%	90.6%	0.6%	0.0%	I	0.07	−0.03
	Q3. chat with others online	6.1%	28.9%	59.4%	4.4%	1.1%	0.0%	M	0.35	−0.89
	Q4. searching for articles	11.7%	2.8%	2.2%	83.3%	0.0%	0.0%	I	0.14	−0.05
	Q5. favorite articles	67.2%	15.0%	16.7%	1.1%	0.0%	0.0%	A	0.82	−0.32
D. Self-media construction information display demands	Q1. time display	13.3%	20.0%	60.0%	6.7%	0.0%	0.0%	M	0.33	−0.80
	Q2. visitors’ statistics	18.9%	19.4%	56.1%	1.7%	3.9%	0.0%	M	0.40	−0.79
	Q3. online population	2.2%	1.7%	1.7%	94.4%	0.0%	0.0%	I	0.04	−0.03
	Q4. popularity display	3.3%	1.7%	1.1%	93.9%	0.0%	0.0%	I	0.05	−0.03
	Q5. latest article	12.8%	14.4%	45.0%	27.8%	0.0%	0.0%	M	0.27	−0.59
	Q6. links to other media	7.8%	2.2%	1.7%	88.3%	0.0%	0.0%	I	0.10	−0.04
	Q7. popular articles	43.3%	22.2%	17.2%	17.2%	0.0%	0.0%	A	0.66	−0.39
	Q8. related information	8.3%	1.7%	1.1%	88.9%	0.0%	0.0%	I	0.10	−0.03
	Q9. Q & A	6.1%	1.7%	1.7%	41.1%	49.4%	0.0%	R	0.15	−0.07

## Data Availability

Data can be acquired from the corresponding author according to reasonable request.
